# The Role of Working Animals and Their Welfare Issues in Ethiopia: A Systematic Review and Meta-Analysis

**DOI:** 10.1155/vmi/7031990

**Published:** 2024-12-23

**Authors:** Tarekegn Derbib, Getasew Daru, Senait Kehali, Sinkie Alemu

**Affiliations:** ^1^Department of Animal Science, College of Agriculture and Natural Resources, Mekdela Amba University, P.O. Box 32, Tulu Awuliya, Ethiopia; ^2^Department of Rural Development and Agricultural Extension, College of Agriculture and Natural Resources, Mekdela Amba University, P.O. Box 32, Tulu Awuliya, Ethiopia

**Keywords:** equine, Ethiopia, food security, livelihood, welfare, working animals

## Abstract

Working animals provide a critical socioeconomic role for many low-income communities, especially in Ethiopia nearly all draft power for agriculture production is generated from working animals. However, the welfare status of working animals remains worst in the country. Hence, understanding the major welfare problems of working animals is the key to improve their welfare status and to maximize their economic contribution. This systematic literature review encompasses 28 studies published between 2010 and 2024 that address the role of working animals and the factors impinging on their welfare. In this review, PRISMA flow diagram was used for literature inclusion and exclusion. In this review, we provide the role of working animals on improving the livelihood of peoples. According to the review, working animals were used as a direct food source, served as draft power, as income source, and also used for other purposes. This review also indicates low treatment to disease and injury, poor access to feed and water, no freedom from beating and distress, low access to safe shelter, and no freedom to express normal behavior are the major welfare problems in Ethiopia. The review also finds the gaps in animal welfare measurement and data analysis method, which should be examined by future researchers.

## 1. Introduction

Working animals support the livelihood of more than one billion people that comprise the world's poor and provide a crucial role in the achievement of sustainable development goal (SDG) by enhancing access to education, reducing the impact of climate change, enhancing climate people's resilience, reducing the burden of women, and allowing water shortage [1–4].

Particularly in the agricultural sector, working animals offer more than 50% of the world's agrarian energy as working power, whereas other human-made engines provide less than 30%, with the remaining percentage provided by human force [5]. In addition to these roles, livestock are mainly considered as livelihood assets and social safety-net for poor farmers, particularly for women and pastoralist farmers [6, 7].

Beyond agricultural sectors, in many developing countries such as India, Pakistan, and other sub-Saharan African countries, the increment in fuel price causes several urban workers to shift from motor vehicles to working animals especially equines for transportation [8]. This idea is the ground reality in our country Ethiopia, where working animals are still crucial and are the major source of income for their owners [9, 10]. Even though it varies based on the location, the most common transport animal in Ethiopia includes use of donkeys, camels, horses, and mules as pack animals, for pulling carts and for riding [2, 11]. According to the report of Brooke [12], more than one million cart donkeys and 250,000 cart horses are serving millions in different parts of the country. In addition, cattle provide nearly all the draught power for agricultural crop production at the smallholder level in Ethiopia [13].

According to The Donkey Sanctuary in Ethiopia [14], Ethiopia is the largest working animal husbandry that comprises 12.4 million oxen, 5.7 million donkeys, 2.4 million camels, 2 million horses, and 0.3 million mules. All these working animals play an indispensable role in improving food security and diminishing poverty. It is not exaggerated to say that the economy of the country highly relies on the livestock sector, and it contributes to agricultural gross domestic product and foreign exchange through exports of animals, meat, leather, other animal products [15, 16].

The issue of animal welfare has a multidimensional face which includes economic, social, political, ethical, and moral dimensions [17]. However, evidences indicate that [18–20], despite working animals provide a significant role in the improvement of the communities' livelihood and the entire economic growth, the attention given to protect their welfare is very low in Ethiopia, due to that they suffer from several diseases, physical and mental injury, overworking, low health care, and shortage of feed that lead to poor work performance [21–24].

Several literature studies were attempted to detect the key health and welfare problems of working animals in Ethiopia [11, 25–28]. The main focuses of the previous literature are different, for instance, Kumar et al. [29], Birhan et al. [30], Molla et al. [31, 32], Fsahaye et al. [33], and Bereket and Addis [34] investigate the health and welfare status of working donkeys in different parts of Ethiopia. The other literature studies such as Tadesse [35], Asmare and Yayeh [36], Chala et al. [37], and Merridale et al. [10] looked at the health and welfare status of cart-pushing horses. Studies by Solomon et al. [38], Ali et al. [39], Getnet et al. [40], and Hundie et al. [41] mainly focus on the health and welfare status of cart-pulling mules. Furthermore, the contribution of working animals to improve the livelihood and food security of smallholder farmers has not been perceived rightly and is less valued in the economy of Ethiopia [11, 21, 28, 42]. Even though these literature studies fail to show the comprehensive welfare status of working animals and their socioeconomic importance, their studies focus on a specific area, specific welfare parameters, and single working animal. This needs a comprehensive understanding of working animals' welfare and their role in people's livelihood.

Therefore, the main aim of this paper is to systematically review the welfare status of working animals and their role in livelihood, food security, and agriculture production in Ethiopia. By doing so, this systematic review contributes to the literature in two aspects; first, this review provides comprehensive information and knowledge on the general welfare problem of working animals by integrating the literature published previously, thus facilitating the refining of effective strategies to improve the welfare status and economic return of working animals. Second, this review discusses the research gaps in the reviewed literature in measuring the welfare status of working animals. This helps to inspire future researchers and policy designers regarding working animal welfare.

### 1.1. Objective of the Review

1. To review the role of working animals on the livelihood, food security, and agriculture production
2. To review the major welfare problems of working animals in Ethiopia
3. To review the gaps on the previously published literature

## 2. Methodology

For the achievement of our review research objective, the researchers apply a systematic review method starting from the literature searching up to the end of writing and structuring of the review report. This review used previously published and unpublished secondary data such as books, research articles, reputable journals, annual reports of national and international organizations, policy briefs, and other indexed scholarly materials that are related to the topic of this review's research. The literature search was carried out from December 2023 until April 2024.

### 2.1. Literature Searching Technique

To collect the literature studies that are used for this review, the researchers apply different search methods from different databases including, Google Scholar, Scopus, Web of Science, and other best sources of information for this review. After identifying the sources, the researchers develop the search criteria that is the literature must be published and documented between 2010 and 2024. The scope of the literature is limited to Ethiopia or studies that comprise Ethiopia. The studies were identified using keywords such as working animals, equines' welfare, cattle welfare, animal welfare, working animals and their role in achieving livelihood, food security, and agriculture production in Ethiopia.

### 2.2. PRISMA Flow Diagram Description

#### 2.2.1. Literature Screening

**Figure 1:**
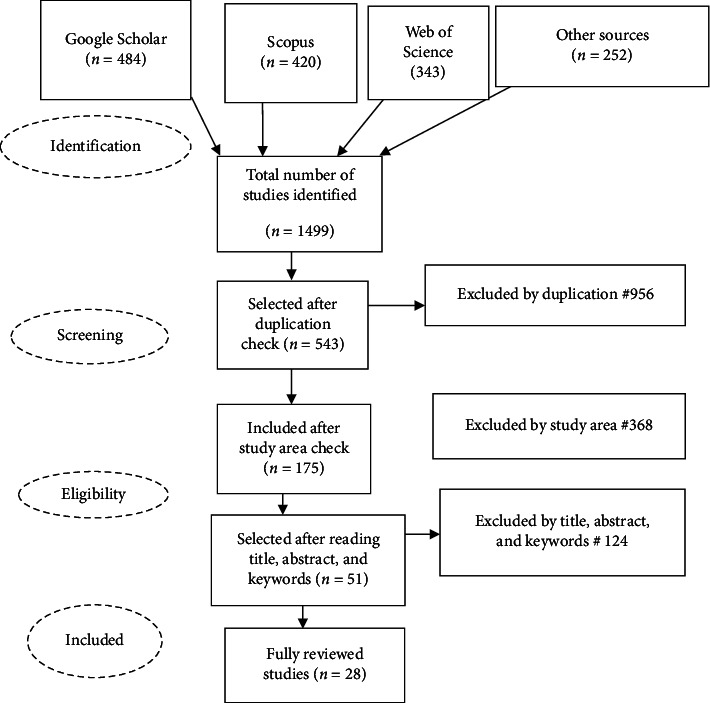
Graphic representation of the overall searching and screening process of this review.

The literature inclusion and exclusion criteria were developed by considering different principles that assure the appropriateness of the literature to achieve the review objectives. Based on searching different databases including Google Scholar, Scopus, Web of Science, and other sources, a total of 1499 studies were obtained. However, based on various inclusion and exclusion criteria, only 28 literature studies were selected for full reading and a deep review of the topic.  Figure 1 shows the overall searching and screening process of this review.

## 3. Results

### 3.1. Extracted Data From the Literature

As shown in Table 1, in this systematic review, data extraction and classification from the selected pieces of the literature is one of the basic and principal tasks of the review. The data extraction process is carried out based on predefined criteria that include years of publication (2010–2024), study area (Ethiopia), and studies that focus on working animals' welfare and their role in the livelihood of people. Of the selected studies, the majority of them address both working animal welfare and their role in a single study; some of them address the welfare aspects only, and others address the economic role merely, but all of these previous studies are considered in this review.

**Figure 2:**
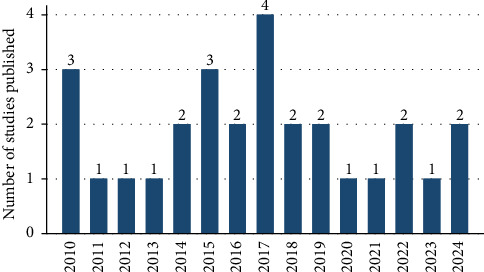
Number of studies published on working animals' welfare and their roles (2010–2024).

Figure 2 depicts the number of studies published on working animals' welfare and their importance on livelihood and food security between 2010 and 2024. According to Figure 1, the maximum number of publications (four publications per year) was achieved in 2017, whereas the minimum number of publications was obtained from 2011 to 2013, 2020–2021, and 2023, with only one publication per year. The other years (2014, 2016, 2018, 2019, 2022, and 2024) indicate that two publications per year were obtained.

This result displayed in Figure 2 implies that the number of studies published on the welfare of working animals was not steadily increasing or decreasing, but it varied over time. Conducting studies are critical to have an in-depth understanding of the knowledge, attitudes, and practices of animal welfare among animal owners. However, the review result implies that the research attention given for working animals' welfare was inadequate and not institutionalized. From this, we can infer that the issue of animal welfare is much neglected by their owners and by the government agencies. Due to the fact that, still now the government of Ethiopia in general and ministry of animal science in particular do not implement the policy of animal welfare in the country.

## 4. Discussion

### 4.1. The Role of Working Animals on Improving Livelihood, Food Security, and Agriculture

Ethiopia has the largest cattle population in Africa, that is, 31 million head [46, 57]. From which 9 to 10 million are used for draught power in agriculture and for other livelihood purposes. In Ethiopian, the majority of smallholder farmers use indigenous Zebu cattle breeds in pairs, and they are primarily used for seed-bed preparation and threshing. On the other hand, horses, donkeys, and mules are used in paired with the same species or with others to plow the crop land in the area where oxen are inadequate [58–60]. More importantly, equine species (horses, donkeys, and mules) are used for transport in most parts of the country. Ethiopia has six donkey breeds, namely, Abyssinian, Afar, Hararghe, Ogaden Omo, and Sinnar, which are distributed in the highland part of the country [61–63]. In Ethiopia, a donkey's significant role is transporting, pulling cart, and plowing purpose [64, 65]. In the lowland and drier regions, camels are also used exclusively as pack and transport animals.

**Figure 3:**
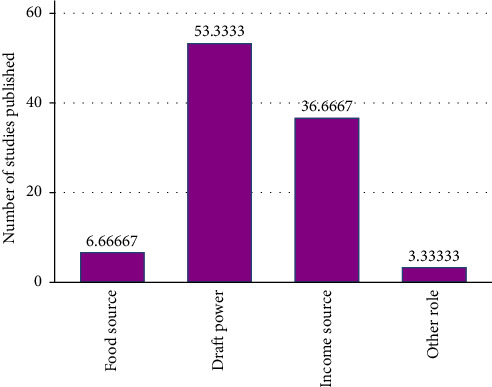
Synthesized result from the literature on the role of working animals in Ethiopia.

As shown in Figure 3, the synthesized descriptive statistics also revealed that, from total reviewed studies, 53.3% of the literature assured the important role of working animals as a daft power, whereas 36.6% of the literature indicates the critical role of working animals as the best source of income and employment creation especially for poor people in rural and urban areas. The other 6.6% of the selected literature displays working animals served as direct food sources, and 3.33% of studies point out other important roles of working animals in the livelihood of the people.

According to the review result, several scholars [15, 54, 66] confirmed that working animals are broadly used throughout Ethiopia and play indispensable roles in everyday activities of people and they generate income for the people that use them. They have direct or indirect linkage with the improvement of households' food security and overall livelihood status.

For example, a study conducted by Asrat et al. [67] showed that cattle are a direct source of food through the provision of meat and milk, and they also play an indirect role in food production as a draft power such as for plowing, threshing, and rarely transportation of products. Ninety percent of rural Ethiopians rely on draft animals, including oxen for various purposes. The other studies by authors in [68–71] indicate that oxen are an important source of income through direct sales of animals, meat, skin, and other products. Oxen can also be rented in cash during the cropping season, and they are an important source of income for farmers, according to Cochet [44]. According to Aune et al. [46], oxen are the primary source of draft power for crop production, but rarely farmers also use cow traction as an alternative to oxen plowing. However, this systematic review assured there was a literature gap on the role of cows as a traction power.

According to Tefera [13], Ethiopia has six million draught oxen, which is equivalent to 500,000 tractors, and an additional six million oxen are required to satisfy the demand since 60% of farmers hold only one ox or none at all. Poor reproductive performance of local cattle, beef fattening, the expansion of meat exports, and absence of animal feed several were among the factors whoich contribute to shortage of draught oxen in Ethiopia. Replacing oxen by tractor is the challenging task, so integrating motorized technology with draft animal technology is a critical option to tackle the problem.

The intensive review of works of the literature also indicates that the camel is one of the working animals that are used as daft power, transportation of people, and packing commodities around harsh environments that are not suitable for other working animals. In addition, they are a considerable source of food in the various arid and semi-arid areas of Ethiopia where other animals are exposed to difficulties, particularly due to the recurrent occurrence of drought. The consumption of camel meat and milk is indeed taboo in the Christian religion, but they are an important source of food in pastoral and agropastoral Muslim communities of Ethiopia [72–74].

Other similar evidence, in the Somali Region, Ethiopia, indicates that working animals (camel, donkey, and cattle) have a great contribution in enhancing households' financial, human, and social capital enrichment. Particularly, cattle and camels are basic sources of meat, milk, and skin for household food security [6]. In line with the above study, FAO [2] also reported that working animals directly play a crucial role in ensuring food security at smallholder farmers' level by providing milk and meat and indirectly through fertilization of farmland and sale of offspring to purchase food items. Especially, in least developing countries such as Ethiopia, the majority of the population depends on animal power as its main energy source.

The review result also indicates that equines play an indispensable role in creating employment opportunities for poor households and basic means to generate income for households. The majority of equine owners confirmed that the net returns from equine use are significantly higher than the total costs; thus, equines are very useful whether it is for exclusive own use or income generation. The evidence also shows that since the majority of the population in Ethiopia is highly dependent on subsistence agriculture, which is highly susceptible to climatic change risk, so equines are the main source for diversification into nonfarm activities [15, 20, 47, 75].

The evidence from Awi Zone, Ethiopia, reported that equines such as horses are important for the livelihood of many smallholder farmers in a country such as Ethiopia, particularly in the study area, Ankesha Guagusa and Banja Shekudad district. The study indicates horses play a versatile role in the farming activities of smallholder farmers including plowing of farmland, transportation of goods, and human transport [36].

### 4.2. The Major Welfare Problem of Working Animals in Ethiopia

**Figure 4:**
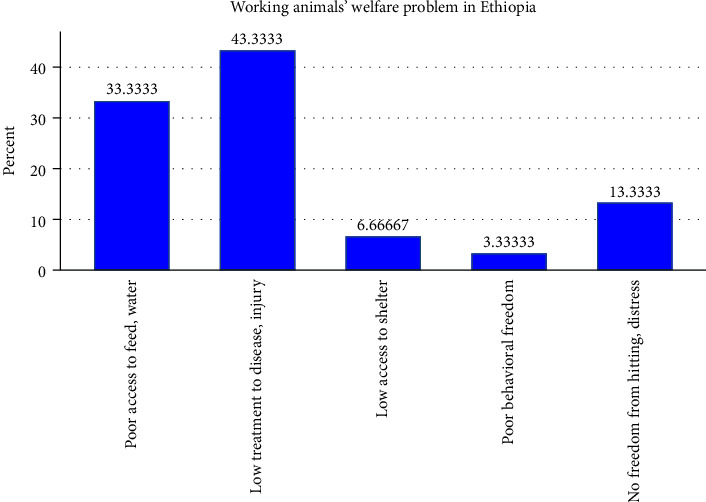
The major welfare problems of working animals in Ethiopia synthesized from the literature.

i. Measuring of working animals welfare: Before talking about the welfare status of working animals, it is basic to consider the indication of farm animal welfare. According to FAWC [76], five major and standardized welfare indicators are used to measure the welfare status of animals. These fundamental animal welfare indicators include “Freedom from hunger and thirst” (access to fresh water and a diet), “Freedom from discomfort” (access to shelter and a comfortable resting area), “Freedom from pain, injury, or disease” (prevention or rapid diagnosis and treatment), “Freedom to express normal behavior” (providing sufficient space, proper facilities, and company of the animal's kind), and “Freedom from fear and distress” (ensuring conditions and treatment that avoids mental suffering).
ii. Working animals' freedom from pain, injury, or disease: As shown in Figure 4, the synthesized result of the literature points out the major welfare problems of working animals reported by previous studies published from 2010 to 2024. According to the result of the review, 43.3% literature studies specify that low prevention and treatment of injury or diseases is a primary welfare problem of working animals in Ethiopia. Previous kinds of literature studies depict that [11, 21, 77], despite equines playing various roles in the community, there is less attention given to their welfare. According to the review result, the majority of equine animal owners do not take their equines to veterinary clinics for treatment, whereas some of the owners use traditional medication to treat them, and only a few owners take them for veterinary treatment. This result depicts that equines were expected to work hard without sufficient veterinary treatment, and when they were sick, they are left to die [78, 79].
  Large bodies of the literature indicate that working animals are highly susceptible to health-related problems, infectious diseases, dermatological diseases, dental problems, musculoskeletal problems, eye problems, wounds, poor breast straps, poor girth, improper harnessing, Hippobosca, and Stomoxys because of their poor access to veterinary treatment [32, 33, 50, 56, 80].
iii. Working animals' freedom from hunger and thirst: The result of this review in Figure 4 also revealed that 33.3% of the working animals in Ethiopia suffered from poor access to feed and water. Most commonly, all equines and very critical donkeys are low-status animals in different areas of Ethiopia because of this they are frequently denied and neglected by their owners to access the kind of feed, water, and healthcare that is made available to other animals such as cattle. This result implied that, despite equines playing fundamental roles in the socioeconomic activities of people, they are perceived as nothing by their owners. This problem leads to no one paying attention to equines for what they eat and drink; their owners remained with them only during work that needs equines' power [11, 15, 18, 19, 34, 43].
iv. Working animals' freedom from fear and distress: Our analysis also indicates that 13.3% of the reviewed literature reported that working animals have no freedom from beating, fear, and distress. Cattle, especially oxen, have more respect for working compared to equines. However, many of the working equines are owned by poor households; hence, ensuring conditions and treatments that avoid mental and physical suffering are ignored [81, 82] Rather, equines are forced to work by beating in the harsh environment, and because of that, most working equines have serious welfare problems, including gait abnormalities, joint swelling, broken skin, deep lesions, and dental problems [18, 42, 49, 50, 80].
v. Working animals' freedom from discomfort: As demonstrated in Figure 4, 6.6% of the reviewed literature reported working animals', particularly equines, low or poor access to shelter and resting areas [33, 52–55]. This indicates that the issue of access to shelter is a serious issue of working animals, especially equines in Ethiopia. Most of the time, cattle have access to shelter because they are high-status animals. However, equines are neglected by their owners and put outside the farmhouse or prepared the poor house for them, this action exposes them to biological, health, physical, and mental problems [11, 43, 83].
vi. Working animals' freedom to express normal behavior: Improving the conditions and facilities for working animals to express their normal behavior is one of the welfare indicators [84, 85]. As shown in Figure 3, only 3.3% of the reviewed literature conducted behavioral assessments on working equines in Ethiopia and assured that there are very limited facilities and situations for equines to express their normal behavior. A significant number of studies indicates that the human–equine relationship is critical to equines' care and for effective utilization of them, but most of the literature indicates that the majority of equines are dull and difficult to catch due to which they are beaten and suffer from chronic fear [33, 52, 55]. The result from Table 2 shows the major working animals in Ethiopia, their major welfare problems for each animal, and the recommended solutions by previous studies.

#### 4.2.1. Farmers' Awareness About Animal Welfare in Ethiopia

The issue of animal welfare existed for a long period of time in Ethiopia, but the country has not formulated regular ways of awareness creation to the public, and the community does not recognize the minimum standards of animal welfare. Despite the country having large number of animals, welfare is not adequately maintained; therefore, low production and productivity are prevalent features of the sector [19].

According to Alemayehu et al. [18], farmers practicing the mixed crop-livestock farming system had better knowledge, attitude, and practice on animal welfare than pastoralist. The evidence indicates that farmers engaged in the mixed farming system had better knowledge on welfare status–related nutrition condition of the animal, animal–human relationship, the importance of water, and health inspection compared to pastoralists. On the other hand, pastoralists had better knowledge on natural animal behavior expression, animal care, and animal suffering than mixed farming communities.

The other evidence confirmed that animal welfare is not only a moral and ethical issue but also a legal one. In Ethiopia, there is only limited source of information, regulation, and guidelines concerning animal welfare at the national level except one course which is given by higher university institution. Due to this, the community is not getting adequate information regarding the welfare situations and protection of animals [94].

### 4.3. Research Gaps

The previous scholar has generally investigated the welfare problem of working animals and their roles on livelihood, food security, and agricultural production. Nevertheless, regarding diminishing the welfare problems and escalating the effect of working animals on food security, additional efforts must be devoted to advance research in the field. For that purpose, this systematic review assembles the research gaps in the existing literature and presents them as follows.

#### 4.3.1. Gaps in the Measurement of Working Animal Welfares

**Figure 5:**
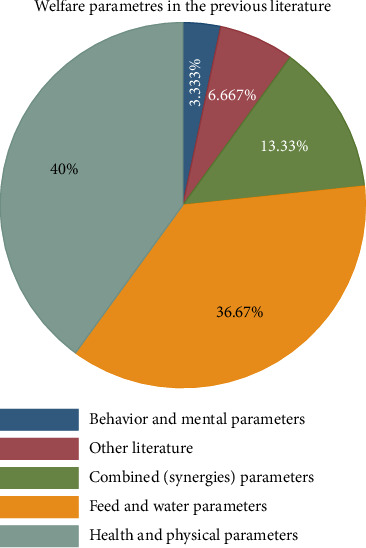
Measurement parameters to animal welfare used in the previous literature.

Applying accurate welfare indicators (measurements) or parameters is a precondition in order to assess and understand the welfare status of working animals. As shown in Figure 5, out of the total reviewed literature studies, only 13.3% of them considered all (combined) parameters in a single investigation, for instance, Girmay [54], Bereket and Addis [34], Aliye et al. [83], and Yalew et al. [55]. However, 40% of the literature only considers the health and physical condition of working animals. The studies conducted by various scholars are witness to this: Scantlebury et al. [95]; Fasil and Yenewhunegnaw [87]; Fsahaye et al. [33]; Molla, Fentahun, and Jemberu [47]; Mathewos et al. [96]. On the other hand, 36.6% of literature studies consider feed and water access of working animals, such as studies by Amenu et al. [97], Asmare and Yayeh [36], and Hadush [98]. However, only 3.3% of literature studies [31, 99] assess the behavioral and mental welfare of working animals. This indicates that the literature considering synergy parameters is important to understand the overall welfare status of working animals. However, as can be seen, the previous literature has not paid sufficient attention to this, and mostly, they used health parameters as overall assessment of working animals' welfare.

#### 4.3.2. Gaps in the Data Analysis Methods

**Figure 6:**
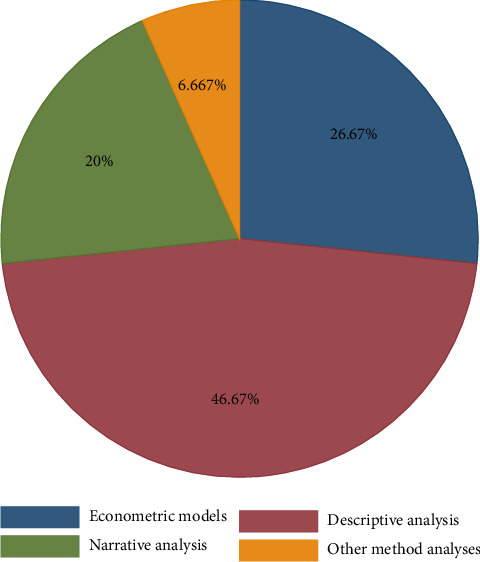
Method of data analysis used in the reviewed literature.

The review results in Figure 6 show a description of the data analysis methods used in the previous literature. According to the review result, 46.6% of literature studies used simple descriptive statistics to analyze the data in their study. While only 26.6% of literature studies used econometric models, and 20% and 6.6% of the previous literature used narration and other method analysis, respectively. Despite selecting the appropriate method of data analysis depending on the nature of the study, it is preferable to apply econometric models that help to identify the relevance and predict the magnitude of the studied welfare parameters to improve the welfare status of working animals. However, the previous literature has given little attention to econometrics and other sophisticated methods of the analysis.

#### 4.3.3. Gap in Studied Working Animals

**Figure 7:**
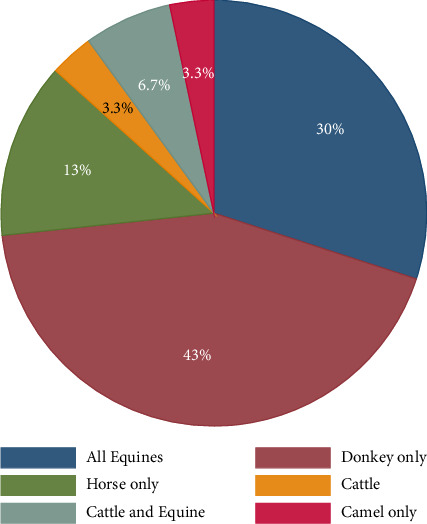
Working animals studied in the previous literature.

As displayed in Figure 7, 43% of previous scholars considered only donkeys in their investigation, whereas 30% of researchers considered all equines (donkey, horse, and mule) in their study. Moreover, 13% of studies consider only horses, while 6.7% of the literature considers both cattle and equine. The result also indicates that only 3.3% literature focuses on cattle and 3.3% on camels. Cattle and camels are among the vital working animals in Ethiopia [6, 46]. Nevertheless, previous scholars gave very little attention to studying the welfare problems of oxen and camels compared to other working equines.

## 5. Conclusion

The main aim of this review was to demonstrate the role of working animals in people's livelihoods, food security, farm production, and poverty reduction to increase the profile of working animals among policymakers and enhance the implementation of policies that secure working animal welfare. The review's findings point out a close connection between human livelihoods, food security, agricultural production, and working animals. However, the welfare issue of working animals has been worse in developing countries such as Ethiopia. To this end, this paper reviewed a range of the literature published between 2010 and 2024. Based on the result of this systematic review, the multidimensional importance of working animals is synthesized into five major roles, including serving as a draft power for the majority of smallholder farmers, as basic income sources, as direct food sources, and other social and cultural roles. Meanwhile, the previous studies documented several welfare problems that negatively impact their working efficiency. These welfare problems are mainly high occurrence of disease and low treatment for disease and injury, poor access to feed and water and no freedom from beating and distress, low access to shelter, and no freedom to express their normal behavior. The review also confirmed that all these problems are emerging from poor awareness about welfare of working animals by their owners. In addition, this paper examined the research gaps in the reviewed literature in the aspects of welfare measurements and data analysis methods. Following the research gap identified by this review, some recommendations are forwarded to concerned bodies. Regarding the increasing food supply challenges, working animals can be used as a fundamental property for sustainable food sources, human livelihoods, and reduction of poverty in Ethiopia. This could facilitate policy-makers and development partners to make these animals a part of national development plans and as part of a holistic approach to ensure food security and poverty reduction. Particularly, all concerned bodies should give especial attention to formulate regular ways of awareness creation to the public about animal welfare standards. Meanwhile, future initiative scholars should strive to investigate the animal welfare status with synergized welfare indicators and rigorous data analysis methods. In this review, we also found it was sometimes difficult to use all welfare indicators in one research than in a single indicator. Hence, researchers should select their own mechanisms that can lead to improve the quality of the research through recruiting appropriate strategies in data collection tools and methods. The following recommendations are forwarded for future scholars: An in-depth exploration of which animal welfare problems are severe in Ethiopia on specified working animals is needed. Further research might compare the intensity of one welfare indicator with others. In addition, significant methodological work is needed on how to robustly capture the impact of animal welfare problem in agriculture and outcomes of animal welfare involvement in the research. It would also be helpful to capture qualitatively the experiences and perspectives of animal owners on protecting the welfare of their working animals.

## Figures and Tables

**Table 1:** Extraction of data from the selected literature about working animals' welfare and their multidimensional roles.

Authors' name and year	Title and study area	Studied working animals	Result of the studies	
			Role of working animals on livelihood, food security, and agriculture production	Welfare problem of working animals
Geiger et al. [11]	Understanding the attitudes of communities to the importance of working donkeys in rural and peri-urban areas of Ethiopia	Donkey	Create economic security, independence, participation in local saving schemes, improve social status, empowerment to marginalized groups, and offer a sense of companionship for poop household	Working donkeys frequently hold low status, and are misunderstood, and given little husbandry and healthcare
Asebe et al. [19]	The general status of animal welfare in developing countries: The case of Ethiopia	Equine and cattle	Not addressed	Less concern about animal welfare and no compressive legislation, rules, or regulations formulated to protect animals' rights
Bereket and Addis [34]	The neglected welfare statue of working donkeys in Ethiopia: The case of Dale district in southern Ethiopia	Donkeys	Not addressed	Feed shortage, poor health, low social status, and low income of owners
Admassu and Shiferaw [15]	Donkeys, horses, and mules—their contribution to people's livelihoods in Ethiopia	Donkeys, horses, and mules	Creation of employment opportunities (gharry and cart services), access to finance (renting equines), local transportation, and other livelihood activities	Feed shortage, poor health, low social status, and poor management
Amante et al. [43]	Health and welfare assessment of working Equine in and around Nekemte Town, East Wollega Zone, Ethiopia	Equine (horse donkey mule)	Not addressed	Feed shortage, traditional health care, wound, overworking, overloading, and housing problems
Cochet [44]	A new perspective on animal traction in Ethiopian agriculture	Oxen	Oxen plays a significant role as a draft power for crop production	Not addressed
Alemayehu et al. [18]	Animal welfare knowledge, attitudes, and practices among livestock holders in Ethiopia	Cattle and Equine	Not addressed	Poor management, nutrition, disease prevention and treatment, responsible care, and humane handling
Mouazen et al. [45]	Improving animal-drawn tillage system in Ethiopian highlands	Oxen	Oxen are the main source of draft power in crop production	Not addressed
Asmare and Yayeh [36]	Assessment on the management of draft horses in selected areas of Awi Zone, Ethiopia	Horse	Horses used for plowing, packing goods, and riding for human transport and play role on livelihood improvement	Lack of grazing land, lack of training, and knowledge on horse feeding management system and disease
Arega et al. [21]	Welfare problems of equines in Sebeta town and suburbs, Central Ethiopia	Equines	Equine has great role in transportation of people and goods	Over work load without sufficient feed, water, and veterinary treatment
Aune et al. [46]	The ox plowing system in Ethiopia: Can it be sustained	Oxen	Oxen and cow used for traction of farming land and crop threshing	Not addressed
Molla et al. [47]	Estimating the economic impact and assessing epizootic lymphangitis in equines in Gondar Zones, Ethiopia	Equine	Equine has an indispensable role to create cart business as an alternative source of income and means of survival for many households to attain their food security	Equine owners had insufficient knowledge and poor control and preventive measures for diseases
Herago et al. [9]	Assessment on working donkey welfare issue in Wolaita Soddo Zuria District, Southern Ethiopia	Donkey	Not addressed	Poor health care, overworking, and overloading
Birhan et al. [30]	Incidence of wound and associated risk factors in working donkeys in Yilmana Densa District	Donkey	Not addressed	Prevalence of donkey wound in olds and adults than youth
Molla et al. [31]	The welfare, watering, housing, feeding, and working management of working donkeys in and around Hawassa City, Southern Ethiopia	Donkey	Not addressed	Skin problem, behavioral change, external wounds, and sores
Chala et al. [37]	Prevalence of work-related wound and the associated risk factors in cart horses in Bishoftu Town, Central Ethiopia	Horse	Income source via transporting services and employment creation for poor households and youths in the area	Wound, improper harness and saddle, poor breast strap, poor girth rope, and infectious diseases
Nejash et al. [48]	Prevalence and associated risk factors of equine wound in and around Asella Town, South Eastern Ethiopia	All equines	Not addressed	Harnessing and infectious diseases
Tesfaye et al. [32]	Study on the health and welfare of working donkeys in Mirab Abaya District, Southern Ethiopia	Donkey	For transportation of commodities and agricultural inputs and outputs	Musculoskeletal, dermatological diseases, eye problem, and dental problems
Ayalew et al. [49]	Monitoring of body weight, body condition, and observation of wound on working equines in Ethiopia	All equines	Not addressed	Injury, improper harnessing, and overworking
Mekete [50]	The prevalence of work-related wound and associated risk factors in working equines: Journal of Medicine and Healthcare	All equines	Not addressed	Prevalence of external injuries
Mekuria and Abebe [51]	Observation on major welfare problems of equines in Meskan District, Southern Ethiopia	All equines	Not addressed	Limited feed, water, and shelter
Usman et al. [52]	Health- and welfare-related assessment of working equines in and around Batu Town, East Shoa, Central Ethiopia	Equine	Not addressed	Harnessing problem, overloading, wound, overworking, disease, lack of veterinary service, and lack of sufficient feed
Mekonnen and Channe [53]	Management practices of working donkeys in urban and rural areas of Assosa District, Benishangul Gumuze region, Ethiopia	Donkey	Assisting livelihood via transportation of people, pack materials, and serving a source of income for poor farmers	Feeding, housing, and health and sanitation problems
Girmay [54]	Health and welfare assessment of working donkeys in and around Axum Districts, Tigray Regional State, Northern Ethiopia	Donkeys	Donkeys play an important role in the transportation of farm produce from the field to home and to markets	Wound, no treatment, no water, and appropriate shelter
Yalew et al. [55]	Assessment of community-based intervention approaches to improve the health and welfare of working donkeys , Southern Ethiopia	Donkey	Not addressed	Prevalence and severity of lameness and wounds on donkeys
Fsahaye et al. [33]	Health and welfare assessment of working donkeys in and around Rama Town, Tigray, Ethiopia	Donkeys	Donkeys provide draft power in crop production, transport services at low cost, and source of income generation	Presence of wound and parasitic and behavioral problems
Chalchisa et al. [56]	Biting flies and associated pathogens in camels in Amibara District of Afar Region, Ethiopia	Camel only	Not addressed	Hippobosca and Stomoxys are biting flies that affect camels' health and body condition
Gina et al. [6]	The role of working animals toward livelihoods and food security in selected districts of Fafan Zone, Somali Region, Ethiopia. Transport, 33	Camel, equine and Cattle	They play a vital role as a source of income, food, and as draft power for different activities	Not addressed

**Table 2:** Summary of working animals, the dominant welfare problems, and possible solution.

Working animals in Ethiopia	Dominant welfare problems for each working animal	Recommended solutions to reduce welfare problems	Reference
Donkey	- Improper harnessing - Skin coat, wound, musculoskeletal, parasitic, ocular, and behavioral problems - Poor animal handling practices and poor attention to donkeys - Feed and water shortage -Limited veterinary support	- Raising awareness of donkey welfares to users - Context-specific welfare improvement interventions - Training and extension service to donkey owners	Lemma et al. [81] Fsahaye et al. [33] Herago et al. [9]

Mule	- External injuries of mules caused by harness problem - Give little care and attention as animals compared to other farm animals - Acute lameness and poor harness quality	- Community education on injury mitigation schemes - Extensive awareness creation for cart mule owners and drivers - Improvement of modern health-seeking behavior of owners - Replacement of poorly designed harnessing materials	Getnet et al. [40] Ali et al. [84] Tilahun et al., [86] Amante et al. [43]

Horse	- Feed shortage, poor health care, lameness, wound, overworking, overloading, and housing problems	- Raising awareness of animal welfare within rural communities - Farmers should take care of their horses	Fasil and Yenewhunegnaw [87]

Oxen	- Working in stress without feed and water - Feeding disorders including bloating - Suffer from wounds inflicted by whipping, beating and poking, and from infections and bruising under the yoke	- Improve nutrition, health care, breeding, and working condition - Raising farmers' awareness to health problems arising from improper use of oxen	Van Dijk et al. [88]; Bobobee and Gebresenbet [89] Tefera [13] Urga and Abayneh [60]

Camel	- Affected by different diseases (Cephalopina titillator larvae and camel contagious ecthyma) - Overloading, lack of feed and water, and poor attitude of owners - Diseases, poor veterinary service, and lack of attention from government	- Strategic community education should be done in order to improve the management system of the camel - Encourage disease control intervention	Teha et al. [90] Melkamu et al. [91] Megersa [92] Previti et al. [93]

## Data Availability

There are no new data generated for this review study.
